# Medical Physics Practice Guideline 4.a: Development, implementation, use and maintenance of safety checklists

**DOI:** 10.1120/jacmp.v16i3.5431

**Published:** 2015-05-08

**Authors:** E. Fong de los Santos, Suzanne Evans, Eric C. Ford, James E. Gaiser, Sandra E. Hayden, Kristina E. Huffman, Jennifer L. Johnson, James G. Mechalakos, Robin L. Stern, Stephanie Terezakis, Bruce R. Thomadsen, Peter J. Pronovost, Lynne A. Fairobent

## Abstract

The American Association of Physicists in Medicine (AAPM) is a nonprofit professional society whose primary purposes are to advance the science, education and professional practice of medical physics. The AAPM has more than 8,000 members and is the principal organization of medical physicists in the United States.

The AAPM will periodically define new practice guidelines for medical physics practice to help advance the science of medical physics and to improve the quality of service to patients throughout the United States. Existing medical physics practice guidelines will be reviewed for the purpose of revision or renewal, as appropriate, on their fifth anniversary or sooner.

Each medical physics practice guideline represents a policy statement by the AAPM, has undergone a thorough consensus process in which it has been subjected to extensive review, and requires the approval of the Professional Council. The medical physics practice guidelines recognize that the safe and effective use of diagnostic and therapeutic radiology requires specific training, skills, and techniques, as described in each document. Reproduction or modification of the published practice guidelines and technical standards by those entities not providing these services is not authorized.

The following terms are used in the AAPM practice guidelines:

- Must and Must Not: Used to indicate that adherence to the recommendation is considered necessary to conform to this practice guideline.
- Should and Should Not: Used to indicate a prudent practice to which exceptions may occasionally be made in appropriate circumstances.

## 1. Introduction

The overall field of medicine is characterized by highly complex, intense, and dynamic processes, where a multidisciplinary team works together using sophisticated imaging, planning, and delivery systems to provide efficient, accurate, and safe patient treatment. As a result of such characteristics, the practice of medicine is susceptible to errors in judgment, errors in communication, lack of compliance with standard operating procedures, as well as workflow inefficiencies. Other complex environments outside medicine, such as aviation^(1)^ and product manufacturing, have successfully used simple tools to aid in reducing human errors. One of these tools is Checklists. Checklists have been extensively validated in nonmedical and medical fields for many years, and have proven to be an effective tool in error management and a key instrument in reducing the risk of costly mistakes and improving overall outcomes.^2^, ^3^, ^4^, ^5^, ^6^

### 1.1. Goals

The goal of this document is to provide a comprehensive strategy for designing, implementing, using, and maintaining clear and effective safety checklists. It is also intended to provide standard components of checklists that can be used as a template in the development of procedure‐ and clinic‐ specific quality management tools. This document does not define the specific elements of a unique checklist for a specific clinical task or process.

Despite the wealth of experience from other industries such as the aviation industry, a systematic approach for developing checklists in the area of medicine is fairly new. A small number of strategies for designing effective safety checklists in the area of medicine have been published,^7^, ^8^ but there is none tailored for the specific needs, environment, and workflow of diagnostic imaging or radiation therapy.

### 1.2. Scope

We recognize that, given the wide variety of practices and technologies in diagnostic imaging, nuclear medicine, and radiation therapy, it is neither practical nor desirable in this document to provide a rigid set of checklists that must be adhered to. Experience from the aviation industry indicates that effective checklists are “works in progress” that evolve as techniques and technology evolves. Additionally, effective checklists need to fit the needs, workflow, and goals of a specific environment or practice. This document, therefore, focuses on guidelines for development of checklists, rather than rigid recommendations. Future AAPM Task Groups or accreditation organization (e.g., ACRO, ACR, or ASTRO) should consider utilizing the steps and methods presented in this document when developing standardized safety checklists as part of their document.

The scope of this MPPG is limited to:

1.2.1. Providing a few example checklists and checklist components, but it will be made clear that the examples are not intended to be adopted en bloc.
1.2.2. Identifying strategies for maximizing the actual use of checklists in the clinical environment.
1.2.3. Identifying the necessary cultural and organizational shift needed to develop, implement and maintain effective checklists.^9^, ^10^ Among other benefits, addressing this component helps dealing with one of the main challenges of checklists implementation, which originates from the mind‐set of highly trained individuals who have deeply entrenched norms and believe that the use of checklists undermines their expertise, diminishing decision‐making and action to provide effective care.^(11)^
1.2.4. Discussing issues related to implementation and use.

#### 1.3. Intended Users

The intended users of this MPPG are individuals involved in quality and safety management in a clinical setting.

#### 1.4. Acronyms and Abbreviations

- AAPM — American Association of Physicists in Medicine
- AHRQ — Agency for Healthcare Quality
- HDR — high‐dose rate
- ICU — Intensive Care Unit
- Linac — linear accelerator
- PDCA — Plan‐Do‐Check‐Act
- PDSA — Plan‐Do‐Study‐Act
- WHO — World Health Organization

## 2. The Role of Checklists in Error Management

Most human tasks can be classified into two basic categories, depending on the type of behavior needed for completion: tasks requiring schematic behavior, in other words done reflexively or “on autopilot”, and tasks requiring attentional behavior, which need a predefined active plan and problem‐solving skills. Errors can be associated with each type of behavior. Failures of schematic behavior are called slips or omissions and they are associated with lapses of concentration, distractions, exhaustion or burnout. Failures of attentional behavior are called mistakes, often occurring due to lack of experience or poor training but also arising from poor judgment or misunderstanding a situation. In medicine, as in similar areas where individuals focus on task performance and completion, most of the errors fall in the schematic category rather than the attentional category.^(12)^ Checklists provide a framework to manage and reduce the risk of errors originated by slips or omissions. The aviation industry is a prime example of the successful use of checklists.^(4)^ They have learned that when pilots and air‐traffic controllers are provided with, and trained on, the effective use of evidence‐based checklists in an environment that motivates them to follow the checklists every single time, the likelihood of errors and accidents is drastically reduced.

Checklists provide a basic memory guide and back‐up for those tasks that are easily forgotten. In other words, checklists ensure that the basics are not missed (e.g., wrong patient, wrong site, missed bolus, missed electron block), allowing the team to concentrate on the difficult and complex tasks that require full attention.^(11)^ Additionally, checklists provide a communication and workflow process that allows teams or individuals to pause, ensuring they are working together. Properly structured checklists facilitate systematic and consistent care delivery, thus reducing variability and improving performance. Checklists must have the right balance of information and structure in order to support clinical practice without compromising or impeding professional judgment or being overly burdensome.^(13)^ In summary, checklists function as a supporting interface among individuals, and between individuals and their environment, helping to guide a particular workflow or procedure.^(12)^

## 3. Organizational Influences on Checklists

Checklists are a human intervention, requiring a strong organizational and social infrastructure to support them. The underlying organizational component for successful implementation and effective use of checklists as an error prevention tool is the commitment of the department or group to establish and practice a safety culture.

Safety cultures are “characterized by communications founded on mutual trust, by shared perceptions of the importance of safety, and by confidence in the efficacy of preventive measures”.^(14)^ Often, a safety culture is said to have four factors.^(15)^ These four factors are:

- The public and private commitment of upper level management to safety,
- Shared attitudes towards safety and hazards,
- Flexible norms and rules to deal with hazardous situations, and
- Organizational learning.

Bosk et al.,^(10)^ in their article entitled “Reality check for Checklists”, states: “The mistake of the ‘simple checklist’ story is in the assumption that a technical solution (checklist) can solve an adaptive (sociocultural) problem.” Further analysis of the Michigan Keystone ICU program shows that the implementation of checklists was only one of several key elements.^(16)^ The implementation of these key elements in combination with the use of checklists led to the success of the program and a reduction of ICU hospital‐acquired infection rates by 70%.^(2)^ These key elements are:

- Summarizing, simplifying, and standardizing the process,
- Creating internal social networks with shared sense of mission and mutual reinforcement mechanisms,
- Gathering, measuring, and providing feedback on clearly defined outcomes, and
- Developing and supporting a safety culture.

Most importantly, a safety culture is an environment where all individuals are empowered and responsible to stop treatment of a patient for any safety concern without fear of consequence, ridicule, or scorn. Providing a checklist to individuals and teams without building the right environment and organizational support will be a futile effort. Management and leadership support for this process is essential. Incorporating checklists into practices requires strong mechanisms to promote teamwork, support communication, and reinforce training and shared knowledge. Checklists also provide a framework by which any team member can challenge the team authority; in fact, checklists make that challenge an explicit responsibility of those conducting the list. Checklists alone cannot provide enhancements in safety and quality, but in the appropriate organizational environment, checklists can be an exceptional safety management tool.

It is the responsibility of the practice leadership to develop and maintain a safety culture, and the tools associated with that commitment. The support of the department leaders (e.g., physicians, chief radiation therapists, medical physics leadership, chief dosimetrists, administrators) is necessary to help the checklists assimilation process. Leadership must encourage their departments and groups to investigate and develop safety tools, and consider this activity as part of the clinical time allocation for both individuals and teams that would like to start their checklists program.

## 4. Checklist Team — Qualifications and Responsibilities

Staff requirements, time allocation, and resources needed to develop and implement a checklist will scale with the scope of the checklist, as well as the size of the practice where it will be utilized. It can range from one single individual working for a day to a sizable team with member representation from each clinical care group (e.g., radiation therapist, dosimetrist, physician, nurse, physicist) working for several months. Developing a safety checklist for the utilization of a water scanning system for annual quality assurance on a single linac in a practice with only one medical physicist is an example of a setting where there is no need to create a team, and the medical physicist can independently develop such a checklist. As the practice grows or the scope of the checklist is broader, the creation of a team is an essential component and will have a positive impact during all the stages of the checklist development and implementation. The development of a safety checklist used prior to a stereotactic radiosurgery procedure in a large practice with multiple linacs and large clinical groups is an example where the creation of a team would play an essential role on the success of the checklist. Such a checklist might include elements from multiple professional groups, [e.g., verification of patient identification (radiation therapists, nurse, physician), patient consent (physician, nurse, radiation therapist), treatment site (physician, qualified medical physicist, radiation therapist), and dose verification (qualified medical physicist)]. A team approach is critical in the development and use of such a checklist. Additionally, because of the size of the practice, the developing, validation, and implementation processes might take multiple iterations during several months.

Team members who will be participating on the checklists development and implementation processes should possess the technical expertise, knowledge, and experience of the area, process, or procedure where the checklists will be utilized. They should also be empowered to speak directly and honestly about the utility of the checklist, thus avoiding a situation where it will go unused or will only hamper efficiency without improving safety. Checklists have a strong sociocultural component because they rely completely on individuals' motivation, commitment, and intervention to be effective as an error prevention strategy. Therefore, an individual or group embarking on the creation of a checklist will require skills on building teams and collaboration, guiding participation, conducting constructive discussions, and finding and agreeing on mutual purpose, among other management, leadership, and organizational strategies. Too often, these skills are underdeveloped and are not part of any of the team members' formal training. Some recommended literature on this topic can be found on Appendix A.

In summary, defining the team size and time allocation will be driven by both the size of the practice, as well as the scope of the checklist. It is important to emphasize that teamwork is an essential organizational component for a successful checklist when used in large multidisciplinary settings or where the scope of the checklist involves multiple clinical groups. As appropriate, a team approach should be used throughout all the phases of development, implementation, revision, and maintenance of a specific checklist. Additionally, each team member that effectively participates during the development process acquires a sense of ownership, which will have a positive impact during implementation and acceptance of the checklist into the practice.

## 5. Checklist Guidelines

### 5.1. Development and implementation process

**Figure 1:**
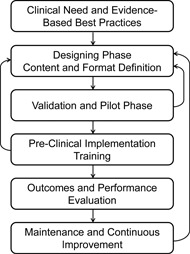
Diagram of end‐to‐end checklist development, implementation, and maintenance process.

Based on current literature and best practices from aviation and medical industries,^1^, ^7^, ^17^, ^18^, ^19^, ^20^ the development and implementation process can be categorized in the following steps (Fig. 1):

#### 5.1.1. Clinical Need and Evidence‐Based Best Practices

The first step in developing a checklist is to find those clinical areas or processes with the strongest evidence to improve quality and safety, and have the highest clinical impact and the lowest barriers for implementation and utilization.^(10)^ Literature review of best practices, empirical evidence, and regulatory, local and community input can help with the selection process. Examples of processes that have shown to be effective quality control checks in radiation therapy and that could benefit from checklists were presented by Ford et.al.^(21)^ and include: physics chart review, physics weekly chart check, and therapy chart review. Additionally, high‐risk and complex procedures are examples of procedures where effective safety checklists have the potential to have a high impact as an error mitigation strategy.

When selecting processes or procedures that will potentially benefit from checklists, it is important to consider that an excessive use of checklists could potentially be detrimental to the practice, leading users and teams to experience “checklist fatigue”. Excessive and uncontrolled use of manual safety tools, like checklists, could make processes unnecessarily inefficient, thus decreasing the reliability of the tool and adding another layer of complexity.^(17)^ With this in mind, the selection process should concentrate on those tasks that are critical, often missed, and overlooked, and can potentially put the patient at the highest risk for harm if not done or missed.^(7)^ Checklists must never be used as a strategy to resolve discipline issues or as a teaching tool by itself. Selecting the right areas and processes that will benefit from using checklists, as well as wisely selecting the checklist elements, are fundamental steps leading to the success of the tool, outcome improvements to translate clinical evidence into practice.

#### 5.1.2. Designing Phase — Content and Format Definition

Poor selection or ambiguity on the checklist goal, role or tasks will most likely lead to failure on the checklist intervention.^(22)^ Therefore, each checklist intervention needs to be associated with an explicit, concise, and unambiguous behavior. The content of the checklist should be organized so it facilitates efficient workflow. The language and sentences used for the checklist items should be simple, direct, and unambiguous, yet maintain the specialized language of the field. Checklists design should incorporate the user or team context, complementing the workflow and avoiding interference with safe and efficient care delivery. The additional time and resources needed to use and perform the checklists should be optimized and factored in the specific workflow. When borrowing checklists from other practices, the content and format of the checklist should not be considered absolute and will need to be evaluated and modified to fit each practice environment and workflow. Checklists should reflect up‐to‐date processes and procedures and reflect the current clinical operational context.

#### 5.1.3. Validation and Pilot Phase

The validation and pilot phases are essential for the success of the checklist and will help the development team detect and identify problems, risks, and issues before clinical deployment, thus avoiding complications that could lead to resistance to using the checklist and the creation of unnecessary barriers. Pronovost and Vohr^(23)^ and Gawande^(11)^ have described and emphasized the importance of the validation and pilot phase and the positive impact on their successful implementation. The aviation industry also relies on this phase before official deployment of their checklists.^20^, ^24^ This step is the first feedback loop back to the designing phase, as shown in Fig. 1. In most situations, the validation of the checklist is a continuous iterative process, requiring several revisions by the development team until the checklist design is acceptable (i.e., it achieves the initial goal and it maintains a satisfactory workflow). During the validation process, the development team works on reaching consensus on the usability, timing, potential risks, team interaction, format, and content of the checklist. After initial validation, the checklist must go through a thorough pilot testing process, if at all possible in a simulated clinical setup, conducted by a group representing the target individuals or team. Depending on the scale of the target group and the scope of the checklist, standard quality control methods like Plan‐Do‐Study‐Act (PDSA), as well as heuristic evaluation using interviews, focus groups in clinical situations, and surveys, are tools that can be used during the pilot phase to collect data and improve the format and conducting method of the checklist.^(18)^

#### 5.1.4. Preclinical Implementation Training

Effective training on the use of the checklist must precede clinical deployment. Target users and teams must have a complete understanding of the purpose and methodology for using the checklist, as well as the goal of each single item on the list. Consistent training should prevent misinterpretation of the items in the checklists and minimize erroneous answers or checks. During the initial time following clinical implementation, the development team should follow and monitor users and teams in clinical situations, provide guidance, and gather data to further enhance the tool.

If it is discovered that the checklist contains faults or anomalies leading to common mistakes or confusion, it is important to correct the problems promptly and, if necessary, loop back to the designing stage for additional improvement of the checklist, as shown in Fig. 1.

In addition, the checklist developers should seek to identify barriers to the use of the checklist.^25^, ^26^, ^27^ These barriers can be classified into the following categories:

- Awareness — staff may not be aware of the checklist; this is usually addressed by training.
- Agreement — staff may not agree with items on the checklist; this is usually resolved with dialogue.
- Ambiguity — staff may not be aware of what the checklist is asking them to do; this is usually resolved with revision of the checklist.
- Ability — staff may not have the resources, time or skills to comply with the checklist; this is usually addressed with changing current clinical processes or developing new ones.

#### 5.1.5. Outcomes and Performance Evaluation

Measuring performance and specific outcomes is the only way to demonstrate that the intervention — in this case, the checklist works. It is advisable to collect baseline measurements pre‐implementation to be able to compare with post‐implementation data and evaluate and quantify the success (or failure) of the checklist. Incident reporting systems provide one method to collect this information.^(28)^ Audits of checklist compliance provide another mechanism to evaluate performance.

Ohri et al.^(29)^ showed that, in clinical trials, radiation therapy protocol deviations are associated with increased risk of treatment failure and overall mortality. Checklists, as an error mitigation strategy and quality assurance tool, have the potential to have an impact on clinical outcomes, but measuring this impact is very challenging and is outside of the scope of the majority of checklist implementation processes. Examples of achievable outcomes and end‐points that should be measured as part of a checklists implementation process include:

- compliance with clinical protocols, procedures, and processes,
- reduction of near‐misses in critical clinical processes,
- enhancement of communication and team dynamic,
- practice standardization, or
- streamline workflow.

Demonstrating, with concrete evidence, the success of a specific checklist will reinforce the utility of the tool to the group and, in some instances, can help motivate skeptical individuals to use the checklist.^(23)^

#### 5.1.6. Maintenance and Continuous Improvement

Checklists should evolve with the practice and reflect the most current, evidence‐based data, published guidelines, end‐user feedback, and organizational changes, as well as updates on internal institutional policies, procedures, systems, machines, and instruments. As part of the practice overall quality assurance or safety program, routine reviews (e.g., annual or semiannual) of the practice checklists, as well as checklist performance and compliance, should be performed. Incident learning systems provide a quality control metric of the checklist performance and can flag when the tool requires further development or possibly additional training on the use of the checklist. A checklist should be considered a constantly evolving document, requiring monitoring and modifications in order to adapt to practice changes.

### 5.2. Approaches to Using Checklists

How a checklist is used depends on its purpose. There are several types of checklists and the variations are discussed below. Some checklists guide the user through a process, preventing the omission of steps. Sometimes the forms also check the data that will go into some process, such as a calculation, or facilitate passing information between team members, such as in a planning directive. Procedures or processes requiring multiple team members to be present at the same time (i.e., SBRT, HDR, SRS, angiogram) might decide to assign one person as the caller/checker of the tasks on the checklists. Upon completion of their corresponding task, the other team members will clearly state their task followed by “check” or “complete”. This approach lets the person calling the task know that the person performing the task heard the call correctly and performed the task. The most suitable method depends on the specific circumstances, the individual versus team approach, and the clinical context where the checklists would be implemented and utilized.

Many check forms are used to intercept possible errors — for example, evaluating a brachytherapy plan before its execution. These forms are often used by a single individual, and are most effective without the participation of the person that originally performed the task.^(30)^ For these forms, where appropriate and without interfering with the workflow, the person doing the check should enter the actual value from the task (such as the dose to the clinical target volume from the plan) and compare it with the corresponding limits (upper and lower limits should be included in close proximity to the relevant item on the checklist). Writing all the values helps the checker notice if the values fall outside the limits. Additionally, performance is also enhanced if the person using the checklist knows that a peer or manager will audit their use of the checklist.

The concept of redundancy is an important factor in the checklists philosophy. In any system where the human plays a central role in the outcome of a process, humans are often the weak link in the system; therefore, it is important to establish parallel redundancy to the human intervention. This principle directly applies to the checklist procedure. Based on the experience from the aviation industry, there are two types of redundancies available for the checklists utilization procedure. The first is between the initial configuration of a system, machine or process and the use of the checklist as a backup only; this is called initial configuration redundancy. The second is the redundancy between team members supervising one another while conducting the checklist; this is called mutual redundancy.^(19)^

More specifically, checklist conducting methods can be classified into four categories:^(18)^

- Static parallel or call‐do. Using this method, the checklist items are performed and completed as a series of read‐do tasks. The checklist leads the process, and directs the team or individual through the process step‐by‐step. In other words, the checklist uses the “cook book” approach. This method does not use any of the redundancy strategies.
- Static sequential with verification. This method only uses initial configuration redundancy, and requires at least two individuals. One person will perform tasks from start to finish. Then, a second team member will verify each item from the checklist. This method is frequently used upon completion of a process (e.g., treatment planning) followed by the independent verification of correct completion of critical items by another team member (e.g., pretreatment plan check).
- Static sequential with verification and confirmation. This method uses a challenge and response mechanism. During processes requiring a group approach, different members of the team perform various tasks. Upon task completion or during a reasonable procedural pause, a designated team member calls the items from the checklist and each responsible group verifies the completion and accuracy of their corresponding tasks. This method uses the combination of initial configuration and mutual redundancies as a safety barrier mechanism.
- Dynamic. This method is suited for complex decision‐making situations, where the team is confronted with multiple options and needs to decide the optimal course of action. Emergency situations or infrequent and unpredictable critical events are suitable for dynamic checklists. This method frequently uses flow charts and workflow diagrams to aid with the decision making process. The aviation industry uses this method for the Emergency and Abnormal Checklists.^(20)^ Recently Arriaga et al.^(31)^ used this method to develop a set of Surgical‐Crisis Checklists.

A summary of the four checklists approaches, with corresponding redundancy strategies and clinical examples, can be found in Table 1.

**Table 1:** Checklist approaches with corresponding redundancy strategies (i.e., initial configuration redundancy or mutual redundancy). The clinical examples provide situations or processes where these approaches can be utilized.

*Checklist Approach*	*Redundancy*	*Example*
Static parallel or call‐do	None (“cook book” approach)	Procedure to set up a water tank
Static sequential with verification	Initial configuration	Plan check process
Static sequential with verification and confirmation	Initial configuration and mutual	SBRT procedural pause
Dynamic	Initial configuration, mutual or “cook book” approach	HDR emergency procedure

### 5.3. Checklist Design Recommendations

The field of Human Factors Engineering uses knowledge about human characteristics, both capabilities and limitations, that are relevant during any design process and aims to optimize the interactions among people, machines, procedures, systems, and environments.^(32)^ There is ample evidence from both the aviation industry and the medical field showing that failing to adequately consider humans in the design and operations of their systems is at best inefficient and at worst unsafe. As mentioned above, the checklist is a tool that relies completely on human intervention for effective performance. Therefore, it is important to consider applying Human Factors Engineering knowledge into the development of checklists.^(33)^ The aviation industry has done this and has developed very effective checklists. The following recommendations have been gathered from well‐established aviation industry guidelines^19^, ^20^ and from multiple disciplines in the medical field.^7^, ^17^, ^18^, ^33^, ^34^ These recommendations can be classified into three main areas: Content; Workflow, Layout and Format; and Physical Characteristics.

#### 5.3.1. Content

- A clear and unambiguous title that reflects the objective of the checklist should be defined.
- Clear guidance on the type of checklist and on what, when, and who is responsible for carrying out each of the actions and tasks in the checklist should be provided.
- Know the task and consider all task scenarios. Process mapping can facilitate understanding all the steps in the process.^(35)^
- Address how the task is, or should be, actually performed.
- Use standard and unambiguous language and terms.
- Consideration of the minimum number of actions that need to be included on the checklists, that will provide an effective and safe patient care, should be utilized in time‐constrained clinical situations and processes.
- Differentiation of automated subtasks from those that require attention must be done. For an automated task, the checklist should include a determination that the task is completed.
- Specific values should be recorded on the checklists if compatible with the workflow.
- The date of creation or last revision of the checklist must be clearly identified.
- All documents should identify the originator and approval route.

#### 5.3.2. Workflow, Layout and Format

- Sequencing of checklist items should follow the clinical process or procedure, thus preventing users from deferring checking items and potentially forgetting or missing those items and tasks.
- When compatible with the clinical process or procedure, the most critical items on the section of the checklists corresponding to that clinical process or procedure should be placed at the beginning of the section and should be completed first.
- Checklist procedures must be compatible with the operational context, restrictions, and needs of the environment where they will be used.
- Situations or processes requiring long checklists should be divided and grouped into smaller sections. Each section can be associated with systems, functions or subprocesses.
- For team‐based checklists, the addition of a completion call (e.g., “checklist complete”) when the checklist is completed should be included. This step provides a cap to the checklist process and enables the team to mentally move from the checklist to other clinical operational processes and tasks.
- Natural breaks and pauses in the workflow, if such occur, should be utilized to perform the checklists.
- An appropriate amount of time to perform each check should be allocated as part of the clinical process or procedure. Studies show a negative relationship between the speed of performing the check and the accuracy of the check.^(36)^
- Standardization of the format, layout, presentation, and the checklist process should be utilized, especially if multiple checklists are utilized in a group or practice.
- Distractions and unnecessary interruptions during the performance of the checklist should be minimized.
- Fatigue (particularly mental, but also physical) should be minimized. The process should include pauses if appropriate or needed.
- The form should be quick and easy to read.
- A useful checklist must be simple but thorough.
- Utilization of checklists should be part of Standard Operating Procedures of the practice.
- When compatible with the clinical process or procedure, checklist items aimed at improving the communication among team members should be included.
- Revision to the checklist should be made as appropriate based on concerns raised by those using the checklists. For example, use of the checklist may introduce new risks.

#### 5.3.3. Physical Characteristics

- Font types that have clear differentiation between characters (e.g., Sans‐serif fonts, Helvetica, Gill Medium or Arial) should be used.
- Font type should be consistent throughout the checklist.
- Lower case with upper case initial capitals should be used. Use of upper case should be limited for checklist and section headers.
- Italics for comments, notes, or supporting information are acceptable, but it should be used sparingly.
- A font size that it easy to read at about arm length (60 cm) should be used. (This is especially important for paper‐based checklists used under dim light conditions).
- Font size for headings should be 14 pt (with a minimum of 12 pt).
- Font size for normal text should be 12 pt (with a minimum of 10 pt). For cases where a checklist needs to be contained on one page, font size smaller than 12 pt may be appropriate, but must never be smaller than 10 pt.
- Black text on a white or yellow background should be used, with white text on a black background as an acceptable alternative.
- Colored text should be used only with caution because of difficulties in reading colors in some lighting conditions and also because of the possibility of causing confusion among colorblind individuals. Colors can be useful to differentiate tasks or personnel assignments, but should be used after other methods have been exhausted. Knowledge of the environment, as well as the limitations of the people using the checklist, dictate whether using color differentiation will be appropriate.
- Pastel shading can be used effectively to discriminate specific items on the checklist (e.g., cautions, consequences), but they should be used sparingly.
- The following are effective highlight methods for situations or items that require a special emphasis and differentiation, but should be used sparingly to maximize the effect:
  ○ bold type
  ○ larger font size
  ○ underlining
  ○ boxing text on a white or colored background
- Pink or red pages should not be used.

**Figure 2:**
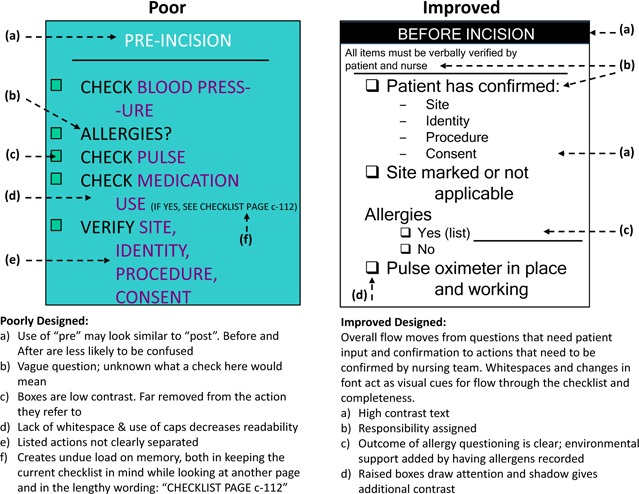
Visual comparison between a poor and an improved checklist (with permission from Dr. McLaughlin and AHRQ WebM&M: http://www.webmm.ahrq.gov/perspective.aspx?perspectiveID=92)

Using some of the concepts and suggestions previously described, Fig. 2 shows a visual comparison between a poor and improved checklist.^(33)^ Appendix B contains examples of clinical checklists use in radiation oncology, diagnostic imaging, and other areas of the medical field.

In addition to the items listed above, consideration should be given to the technical implementation of the checklist. Electronic systems have several potential advantages over paper‐based implementations including the possibility of electronic interlocks such that a treatment cannot proceed if the checklist is not complete and the potential to perform quick audits of checklist conformance. An electronic‐based checklist, however, can have strong disadvantages when not implemented well. Electronic documents can be notoriously challenging in some electronic medical records. They may also serve to tie at least one user to a computer terminal. These disadvantages are enhanced when the checklist is used in a time‐critical procedure. Pilot testing is a valuable method for uncovering such potential problems. Implementation on portable electronic devices may serve to address some of these issues in the future.

## 6. Conclusion

Effective checklists support human thinking, allow constructive team member interactions, and facilitate a systematic care delivery by reducing process variability. Developing and implementing successful checklists require a strong organizational and social infrastructure, as well as the application of well‐defined human factors engineering concepts. The guidelines presented here summarize the evidence and knowledge of the aviation industry and other medical disciplines, and are aimed to guide teams and individuals in our field to develop, implement, and use checklists as a robust and effective error mitigation strategy.

## ACKNOWLEDGMENTS

This guideline was developed by the Medical Physics Practice Guideline 4 task group of the Professional Council of the AAPM.

MPPG 4 group members:

Luis E. Fong de los Santos, PhD, Chair

Suzanne Evans, MD

Eric C. Ford, PhD,

James E. Gaiser, PhD

Sandra E. Hayden, MA, RT (T)

Kristina E. Huffman, MMSc

Jennifer Lynn Johnson, MS

James G. Mechalakos, PhD

Robin L. Stern, PhD, FFAPM

Stephanie Terezakis, MD

Bruce R. Thomadsen, PhD, FAAPM

Peter J. Pronovost, MD, PhD, FCCM, Consultant

Lynne A Fairobent, AAPM Staff

AAPM Subcommittee on Practice Guidelines — AAPM Committee responsible for sponsoring the draft through the process:

Russell Tarver, MS, Chair

Maria F Chan, PhD, FAAPM, Vice‐chair Therapy

Jessica B. Clements, MS

Jonas D Fontenot, PhD

Luis E Fong de los Santos, PhD

Arthur J Olch, PhD, FAAPM

Joann I Prisciandaro, PhD, FAAPM

J Anthony Seibert, PhD, FAAPM, FACR

S Jeff Shepard, MS, FAAPM, Vice‐chair Imaging

Jennifer B Smilowitz, PhD

Gerald A White Jr., MS, FAAPM

Lynne A Fairobent, AAPM Staff

## Figures and Tables

**Figure B.1:**
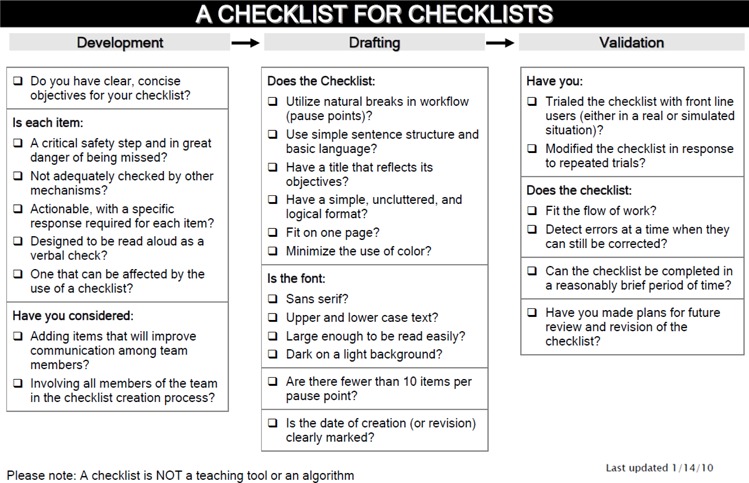
The Checklist for Checklists was created by Dr. Atul Gawande, the Brigham and Women's Hospital Center for Surgery and Public Health Dissemination Team, and Dan Boorman of Boeing. The goal of this checklist is to aid the checklist creation process. A pdf copy of this checklist can be found on: http://www.projectcheck.org/checklist‐for‐checklists.html_ (with permission from Dr. Atul Gawande and the Safe Surgery 2015 group at Harvard School of Public Health).

**Figure B.2:**
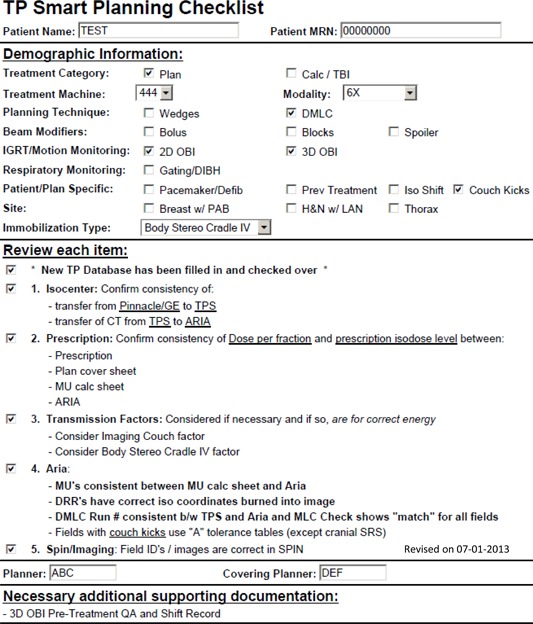
This is an example of a smart checklist tool for the treatment planning process. This tool was developed by James G. Mechalakos, PhD., Sean Berry, PhD, and the group at Memorial Sloan‐Kettering Cancer Center. Based on the plan demographic information and specific treatment procedure, the smart checklist tool automatically generates a custom‐built checklist. This has the benefit of avoiding items that are unrelated to the current plan, so the checklist is shorter. The tool incorporates certain forcing functions such as entering “Covering Planner”, which is a required field for the checklist to be printed. (Courtesy of James G. Mechalakos, PhD.)

**Figure B.3:**
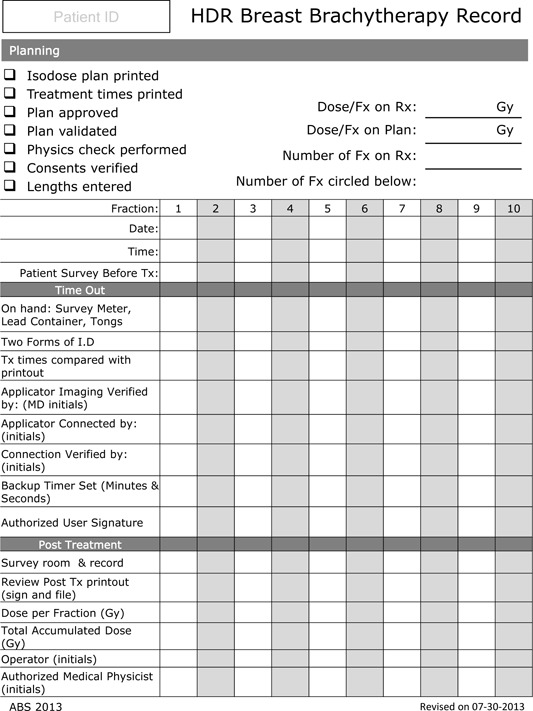
Example of checklist for High‐Dose‐Rate (HDR) Breast Brachytherapy Record proposed by the American Brachytherapy Society (Courtesy of Bruce Thomadsen and Zoubir Ouhib).

**Figure B.4:**
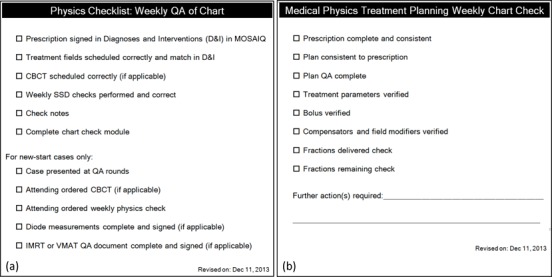
(a) Example of a checklist used as part of the physics weekly chart check process from the University of Washington (Courtesy of Eric C. Ford, PhD). (b) Example of a checklist used as part of the physics weekly chart check process from Yale University Cancer Center (Courtesy of Suzanne Evans, MD). Conceptually Figs. B.4(a) and (b) are achieving the same goal; nevertheless the specific elements and organization of the checklist items will depend on the clinical setting, workflow, and needs of each practice. These examples emphasize the fact that each practice needs to go through its own implementation and validation process to make sure that a checklist would meet its needs.

**Figure B.5:**
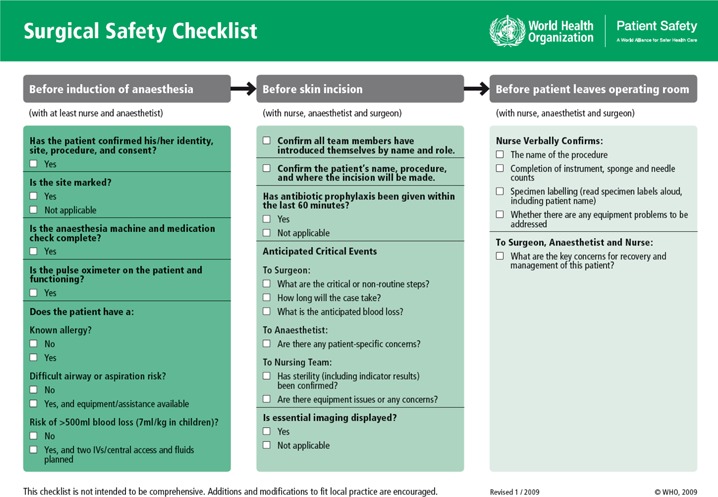
Developed by the WHO and collaborators at Ariadne Labs (http://www.ariadnelabs.org), this checklist identifies key safety steps during perioperative care that should be accomplished during every single operation no matter the setting or type of surgery. More information and details of this checklist can be found on: http://www.safesurg.org.

**Figure B.6:**
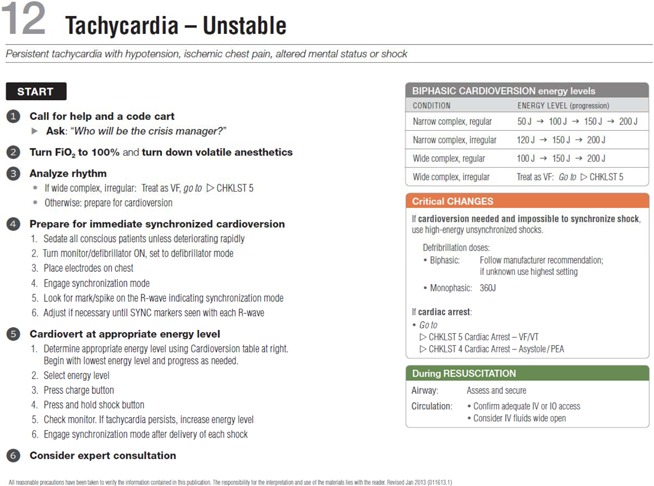
This checklist is one of the 12 checklists developed by the team at Ariadne Labs (http://www.ariadnelabs.org) with the goal of improving care during the most common operating room crises. This checklist shows the differences in layout and approach when developing and using checklists for emergency or crisis situations. For more information on this project follow this link: http://www.projectcheck.org/crisis.html. (With permission from Dr. Atul Gawande and the Safe Surgery 2015 group at Harvard School of Public Health).

**Figure B.7:**
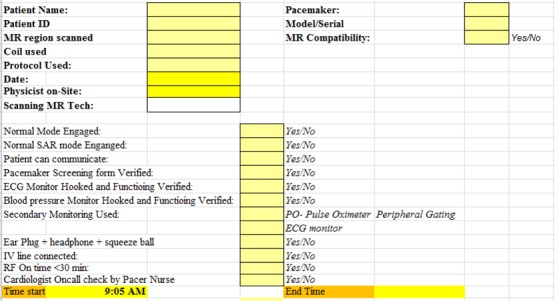
Pre‐MRI procedure medical physicist checklist. This is an example of a checklist used in diagnostic imaging. This checklist was developed by Anshuman Panda, PhD and Judy R. James, PhD from the Diagnostic Radiology group at Mayo Clinic Arizona. The goal of the checklist is to manage patients with pacemaker devices that will have an MRI procedure. (Courtesy of Anshuman Panda, PhD)

## APPENDIX A: Suggested Literature in Organizational Work, Leadership, and Team Building

- Goleman D. Leadership: the power of emotional intelligence, 1st ed. More Than Sound; 2011.
- Goleman D, Boyatzis RE, McKee A. Primal leadership: learning to lead with emotional intelligence. Boston: Harvard Business Review Press; 2004.
- Patterson K, Grenny J, Maxfield D, McMillan R, Switzler A. Influencer: the power to change anything, 1st ed. New York: McGraw‐Hill; 2007.
- Patterson K, Grenny J, McMillan R, Switzler A. Crucial conversations: tools for talking when stakes are high, 1st ed. New York: McGraw‐Hill; 2002.

## APPENDIX B: Examples of Checklists

B.1, B.2, B.3, B.4, B.5, B.6, B.7 are examples of checklists use in radiation oncology, diagnostic imaging, and other medical field areas, as well as a checklist for checklists created by Dr. Atul Gawande, the Brigham and Women's Hospital Center for Surgery and Public Health Dissemination Team, and Dan Boorman of Boeing.

Additional examples can also be found on the following sites:

- Project Check.— Website designed to provide the public with easy access to a number of life saving medical checklists:
  ○ http://www.projectcheck.org/
- Safesurg.org.— Website designed to support individuals and institutions interested in improving the safety of surgical practices:
  ○ http://www.safesurg.org/

**Disclaimer**: Checklists provided in this document must be used only as examples. We do not advise readers to apply these checklists directly on their practice, unless they perform the corresponding validation to demonstrate that they are safe and useful in their own practice.
